# Hypernatural Monitoring: A Social Rehearsal Account of Smartphone Addiction

**DOI:** 10.3389/fpsyg.2018.00141

**Published:** 2018-02-20

**Authors:** Samuel P. L. Veissière, Moriah Stendel

**Affiliations:** ^1^Department of Psychiatry, McGill University, Montreal, QC, Canada; ^2^Department of Anthropology, McGill University, Montreal, QC, Canada; ^3^Raz Lab in Cognitive Neuroscience, McGill University, Montreal, QC, Canada; ^4^Culture, Mind, and Brain Program, McGill University, Montreal, QC, Canada

**Keywords:** smartphone addiction, social neuroscience, evolutionary anthropology, predictive-processing, cultural affordances, social rehearsal, hungry ghosts

## Abstract

We present a deflationary account of smartphone addiction by situating this purportedly antisocial phenomenon within the fundamentally *social* dispositions of our species. While we agree with contemporary critics that the hyper-connectedness and unpredictable rewards of mobile technology can modulate negative affect, we propose to place the locus of addiction on an evolutionarily older mechanism: the human need to monitor and be monitored by others. Drawing from key findings in evolutionary anthropology and the cognitive science of religion, we articulate a *hypernatural monitoring* model of smartphone addiction grounded in a general *social rehearsal* theory of human cognition. Building on recent predictive-processing views of perception and addiction in cognitive neuroscience, we describe the role of social reward anticipation and prediction errors in mediating dysfunctional smartphone use. We conclude with insights from contemplative philosophies and harm-reduction models on finding the right rituals for honoring social connections and setting intentional protocols for the consumption of social information.

## Introduction

As this paper was undergoing final review, a new wave of editorials about the noxious effects of smartphone use was sweeping the news. Major Apple shareholders, backed by petitions from customers, were now demanding that the tech giant address the growing problem of smartphone addiction and its impact on children’s development (Kawa, 2018). As cognitive scientists who have studied the impact of the internet on human behavior (Veissière, 2016a,b), our aim is to present a nuanced view of the relationship between mobile information technology and human well-being. While we agree that excessive smartphone use can be detrimental to mental health, we aim to recast current understandings of the mechanisms involved in these addictive patterns in a broader evolutionary focus.

In this paper, we offer the provocative claim that current moral panics over smartphone addiction overlook a factor of fundamental importance: there is nothing inherently addictive about mobile technology. We suggest, rather, that it is the *social* expectations and rewards of connecting with other people and seeking to learn from others that induce and sustain addictive relationships with smartphones. Much has been said about Internet addiction and the new medias and technologies that connect us and make us lonely at the same time, leading to adverse mental health consequences (Twenge, 2017). The deeply prosocial nature of these mechanisms, however, is often understated. Compulsive smartphone use, we claim, is not so much antisocial as fundamentally social. Specifically, we argue that mobile technology addiction is driven by the human urge to connect with people, and the related necessity to be seen, heard, thought about, guided, and monitored by others, that reaches deep in our social brains and far in our evolutionary past.

Smartphones, we claim, provide a potentially unhealthy platform for another healthy impulse. As we will see, they can also enable us to remember and celebrate the role of other people in making us who we are, and help us treasure the bonds that make us a uniquely social species.

In fleshing out the social roots of smartphone addiction – and by extension, of human behavior and well-being – we do not intend to produce a general meta-theory that dismisses other, non-social forms of excessive smartphone use. The hyper-sociality of smart-device addiction, rather, may likely occur on a continuum from the directly social to the indirectly social.

Playing video-games, outsourcing difficult tasks like memorizing schedules or spatial orientation, and having instant access to news and information are among of battery of everyday smartphone functions that are known to be highly addictive (Alter, 2017). At a glance, these domains are not readily apparent as social. From an evolutionary perspective, however, the human capacity to function optimally in any environment (and indeed human intelligence itself) is predicated on having access to a large, cumulative repertoire of contextually relevant cultural information devised by others, and that no single individual could invent on her own, or recreate alone in her own lifetime (Henrich, 2016; Mercier and Sperber, 2017). Seeking news and information, to put it simply, are ways to *learn from others*, and to stay updated on *culturally relevant* events and people. Video-gaming is similarly underpinned by social dimensions that may not be readily visible to users and critics alike. While many video-games involve explicit social rewards from playing online with other users (Snodgrass et al., 2016) other uniquely addictive smartphone games like Candy crush do not. The unpredictable rewards derived from so-called “ludic loops” of increased difficulty (Alter, 2017), as we expand in the Section “Predictive-Processing and Smartphones,” typically activate neurobiological systems that increase reward-seeking behavior and addictions in other domains (West et al., 2015). In the next section, we present findings supporting the hypothesis that most smartphone notifications, from email and texting to social media, modulate addictive behavior through the anticipation of *social rewards.* The rewards derived from playing games, however, are social in more indirect ways. The human drive for gaming and competition, indeed, is also rooted in social evolutionary mechanisms, in which intra- and inter-group competition have helped drive the iterative spread of skill, knowledge, and technology from generation to generation (Bell et al., 2009; Richerson et al., 2016). In seeking to excel at a difficult game, we are rehearsing excellence in particular domains of skill, but also in the domain of social competition itself. Smartphones, as we will argue, provide a hyper-efficient extension of deep evolutionary urges for connection with others, learning from others, but also comparing ourselves to and competing with others.

### The Sociality of Smartphone Use

When it comes to smartphone use, current scientific literature and intuitive wisdom are overwhelmingly pessimistic, warning us of the dangers these new technologies enable. According to current research, smartphone use is associated with depression (Steers et al., 2014; Andreassen et al., 2016), materialism (Lee et al., 2014; Twenge, 2017), and social anxiety (Billieux et al., 2015; Emanuel et al., 2015; Hussain et al., 2017), spawning a generation of anti-social, chronically anxious, self-obsessed ‘zombies’ (Lu and Lo, 2017). While these findings raise important concerns about the ‘dark side’ of smartphone use, they tend to focus on new technologies as the sole locus of addiction and pathology. We propose to bring this problem into a broader evolutionary focus, and will go on to argue that the current ‘smartphone obsession’ is neither grounded in, nor indicative of a paradigmatic shift in the psychosocial context in which human experience is invariably framed. Popular accounts, we argue, miss the mark on a crucially important factor: it is not so much smartphones themselves that are addictive, but rather the *sociality* that they afford. We insist that this drive for sociality is a fundamental feature of human evolution that predates smartphones by hundreds of thousands – by some accounts several millions – of years (Hrdy, 2007). Simply put, smartphone addiction is hyper-social, not anti-social.

There is ample evidence to support the claim that smartphone use is inherently prosocial, and by extension, that this prosociality is a core locus of smartphone addiction. First, the majority of smartphone use is spent on social activities such as social networking, text messaging, and phone calls (Li and Chung, 2006; Lopez-Fernandez et al., 2014). Even less interactive smartphone use, like information seeking or surfing the web, has now become implicitly social: ‘likes’, views, and comments are social indices of prestige and collective attention. Second, individuals who use their devices for primarily social purposes are quicker to develop habitual smartphone use (Van Deursen et al., 2015). These findings suggest that it is not just the smartphone itself that is addictive but rather the—direct or indirect—social interaction it enables.

Gendered dimensions of smartphone addiction provide further clues into its inherent sociality. Current findings in evolutionary psychology and social neuroscience indicate that women are on average more proficient at social cognition and tend to display more prosocial behavior than men (Eckel and Grossman, 1998; Andreoni and Vesterlund, 2001; Meier, 2007; Laasch and Conaway, 2009; Rand et al., 2016; Soutschek et al., 2017; see Espinosa and Kovářík, 2015 for alternate explanations). This gender discrepancy is maintained in smartphone use, with numerous studies showing that women use their phones for social purposes significantly more than men do (Tufekci, 2008; Van Deursen et al., 2015). According to our hypothesis, the prosocial nature of female smartphone use would render females more susceptible to addiction. Recent estimates confirm this view: females are more likely to develop addictive smartphone behaviors, experience more anxiety if they cannot use their smartphones, and feel less in control over checking their phones (Thompson and Lougheed, 2012; Van Deursen et al., 2015).

### Imagined Other Minds Guide Our Expectations

Despite minor gendered differences in social cognition, it is not controversial that humans as a whole are a prosocial species. Beyond amply documented findings in developmental psychology attesting to the intrinsic co-evolutionary links between cognition and sociality (Moll and Tomasello, 2007; Tomasello, 2009; Tomasello et al., 2012), recent research on mind-wandering has shown that a large part of our spontaneous mental lives is devoted to rehearsing social scenarios. A recent large-scale investigation using experience-sampling, for example, demonstrated that nearly half of waking time is spent in mind-wandering episodes unrelated to the task at hand (Killingsworth and Gilbert, 2010). Although science on daydreaming often describes the consequences of a wandering mind (e.g., Mrazek et al., 2013), it is likely premature to believe that a cognitive function that occupies such a large percentage of mental life does not confer some adaptive benefit. To explain the ubiquity of mind-wandering, Poerio and Smallwood (2016) have proposed that the phenomenon is evolutionarily adaptive, serving as a platform for offline social cognition. Supporting this view, research shows that all but a small fraction of daydreaming involves social scenarios (Mar et al., 2012; Song and Wang, 2012). Moreover, mind-wandering and social cognition rely on shared neural activation, whereby the neural activity that occurs during daydreaming significantly overlaps with that of core social processes like mentalizing and perspective taking – the very processes that enable an individual to socially flourish (Poerio and Smallwood, 2016). Recent models on the evolution of depression help confirm this social hypothesis for the mechanisms of ordinary cognition. In a series of influential papers, Paul Andrews and colleagues have argued that ‘depression’ (a disorder characterized by cognitive rumination) confers specific *social* advantages to help keep social problems in mental focus. Again, it is of note that women (who are demonstrably more proficient than men at social cognition) experience depression at much higher rates than men. Andrews and colleagues see this as further evidence that a significant part of mental life is dedicated to rehearsing social scenarios (Andrews and Thomson, 2009; Andrews et al., 2012, 2015). All in all, a growing consensus between developmental psychology, cognitive neuroscience, and phenomenology strongly suggests that humans are almost always thinking about and *through* other people (Frith, 2002; Tomasello, 2009; Mar et al., 2012; Ramstead et al., 2016). The time is ripe, then, to elaborate a generalized social rehearsal theory of cognition. In the following sections, we expand on this theory and apply it to smartphone use.

### Social Media and Internet Notifications as Hyper-Natural Monitoring

In a series of recent papers, Ramstead et al. (2016; see also Ramstead et al., 2017; Veissière, 2017) have described symbolically enriched human worlds as organized landscapes of “cultural affordances” grounded in mutual, recursively nested expectations about shared standards of behavior. ‘Culture’, on this view can be conceptualized as patterned allocations of attention; that is, the practice of selectively paying attention, ascribing meaning, and guiding behavior to certain features of the world according to what we expect others to also expect and pay attention to. While what is made salient through collectively shaped attentional preferences acquires different values and affords different experiences from group to group, the capacity for shared attention extrapolated to large groups of generalized ‘like me’ others is a species-wide disposition – the very disposition, mediated by joint-intentionality, that gives rise to cultural forms of life among Homo Sapiens (Ramstead et al., 2016; Veissière, 2017).

On this view, over the course of normal cognitive and social development, humans learn to see the world through the perspective of other people and intuitively imagine context-relevant agents (usually imbued with prestige) to guide them in their actions (Veissière, 2017). From context to context and moment to moment, we outsource a large part of our thinking, feelings, and decision-making to sometimes explicit, most often implicit scenarios of the “what would so-and-so think, feel, or expect me to do” variety.

This reassuring feeling of being watched and guided by imaginary others has been hypothesized to play an important role in the evolution of cooperation, morality, organized religion, and large-scale social life (Whitehouse, 2004; Boyer, 2008; Norenzayan and Shariff, 2008; Atran and Henrich, 2010; Norenzayan et al., 2013). According to this view, often called the *super-natural monitoring hypothesis*, we fashioned our Gods and Spirits to better flesh out the imaginary agents that guide our ordinary cognition, consciousness, action, and moral attitudes.

Instant text messaging, email, and social media provide a platform for our hungry need to be connected, but also for our need to watch and monitor others, and better still, for our need to be seen, heard from, thought about, monitored, judged, and appraised by others. We might call this the *hyper-natural monitoring hypothesis.*

The prevailing – and hyperbolic – view on smartphone use is that it is a sly weapon, responsible for pandemic-like waves of mass loneliness, anxiety, insecurity, materialism, and narcissism among today’s youth – particularly the so-called ‘digital natives’ born after 1994 (Roberts et al., 2015; Weiser, 2015; Pearson and Hussain, 2015; Twenge, 2017). As Jean Twenge has pointed out in her recent book on digital natives (Twenge, 2017), the advent of electronically mediated childhoods in the West was also concurrent with a general shift in parenting culture, and the rise of so-called ‘helicopter parenting’^1^ in particular. Drawing on extensive survey research, she points out that children and youth born after 1994 spent considerably less unsupervised time socializing with their peers than their forebears, and significantly more time on electronic devices. While precise causality behind these two correlated factors cannot be ascertained, we can only note that youth who otherwise do not interact with their peers “in real life” (*irl* in internet lingo) seek to do so with the means available to their generation. Online-mediated life, more to the point, is always, already real life, and as such, it is inherently social.

What current moral panics about digital media often fail to consider, thus, is that *the desire to see and be seen*, and *judge and be judged* is precisely *about other people*. There is nothing abnormal, as such, about seeking self-worth through other people’s point of view. We propose, thus, to think of this urge as fundamentally normal, and anchored in core mechanisms of social cognition that are distinct to our species. On our social rehearsal and monitoring view, smartphones simply equip us with a novel medium to channel innate human sociality. Their proclivity to induce addiction, in turn, simply points to how much others matter to us and how we want to matter to them.

## Predictive-Processing and Smartphones

If the primary motivation of smartphone use is prosocial, why can this technology lead to such negative outcomes? We turn to the science of addiction to describe how mobile technology in particular has sent us into a vortex of anxiety-inducing, hyper-excited, hyper-monitoring.

### A Brief Venture into the Neuroscience of Addiction

The exact nature and neurochemical correlates of smartphone addiction are currently unknown (Elhai et al., 2017). Key insights from the neuroscience of learning and addiction, however, can offer important insights into our attachment to the strange flickering and buzzing bricks that seem to regulate our lives.

As we have seen, smartphone use is at once constitutive of and constituted by a complex landscape of sociality. This landscape, however, is also modulated by notifications from dozens of applications that deliver beeps and buzzes, mostly to alert us that another human has interacted with us. We should now consider where and how ‘addiction’ fits in this picture. Social interaction (digital or not) activates the dopaminergic reward circuits in the basal ganglia (See Krach et al., 2010 for a review). It is important to note that these same circuits are implicated in addictive drug use (Belin et al., 2009), compulsive video-gaming, and reward-seeking in general (West et al., 2015). These are circuits that are also responsible for associative learning: the process by which an individual learns to associate two stimuli (Hebb, 1976; Seger, 2006; Yin and Knowlton, 2006). For associative learning to occur, an initial exposure to a new stimulus must occur alongside a reflex-eliciting stimulus. With a smartphone, nearly all notifications that the user encounters elicit a social value and thus activate the dopaminergic reward circuit, leading the user to anticipate and seek these rewarding notifications. With each occurrence this link grows stronger, and the user will anticipate and seek these rewarding notifications, paving the road for habitual behavior.

The dopaminergic system regulates two functions that govern addiction: the *anticipation of reward* and *outcome evaluation* (Linnet, 2014). An important finding about dopamine and addiction, however, is that dopaminergic surges typically occur *before the reward*, or more precisely when a cue (e.g., a beep indicating that one can press a lever) signals the reliable delivery of a reward (e.g., from pulling a lever). Because arousal decreases with frequent and predictable exposure, reward anticipation is a much more powerful mediator of strong addictions than outcome evaluations of the stimulus itself (Fiorillo et al., 2003; van Holst et al., 2012). According to this finding, addictions become strongest when we cannot figure out the pattern of when to reliably expect them (van Holst et al., 2012). Behavioral scientists call these addiction-inducing patterns *intermittent reinforcement* or *variable ratio schedules* (Zuriff, 1970). Neuroscientists have identified that a cue triggering a behavior that yields a reward 50% of the time is by far the most anxiety-inducing of delivery schedules. A reward delivered 75% of the time, for example can be reliably expected to deliver *most of the time*. A cue signaling a reward that delivers 25% of the time can similarly be expected *not* to deliver most of the time. Such high-predictability schedules (when the brain can reliably predict what is going to happen) typically trigger low arousal. At a 50% delivery rate, a reward schedule is still predictable enough to be enticing, but unpredictable enough to be anxiety-inducing (Fiorillo et al., 2003).

**FIGURE 1:**
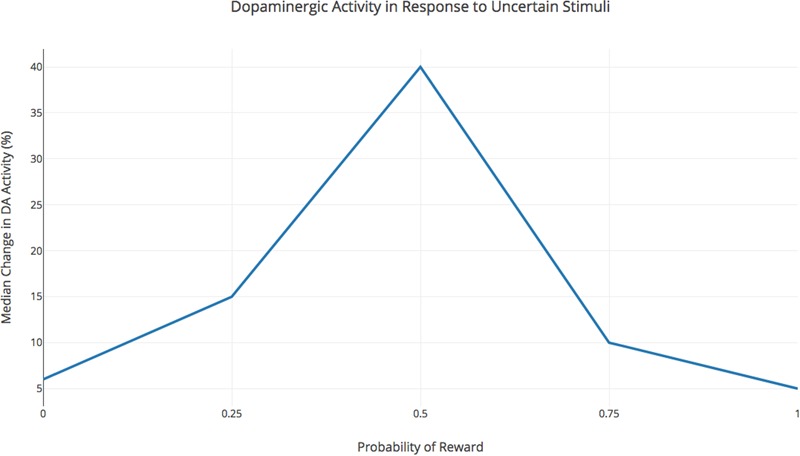
Dopaminergic Activity in Response to Uncertain Stimuli (adapted from Fiorillo et al., 2003, **Figure 3C**). Average Sustained activation of dopamine neurons in a primate as a function of reward probability, whereby the greatest dopaminergic activity occurs when the reward is present half of the time.

The point to take home here is that arousal is more highly correlated with reward anticipation than with the reward itself. When rewards become most unpredictable, in turn, arousal typically becomes negative, giving rise to anxiety (**Figure 1**).

Indeed, the beeps and buzzes of smartphone notifications provide just such an intermittent, variable, unpredictable, but uniquely desirable schedule of rarely met anticipation rewards, thus providing chaotic patterns of reward anticipation that trigger very strong modes of arousal. Because of the deeply social nature of the rewards our phones make us crave, we often become entrenched in vicious cycle of addiction (**Figure 1**).

### Cravings as Prediction Errors

**FIGURE 2:**
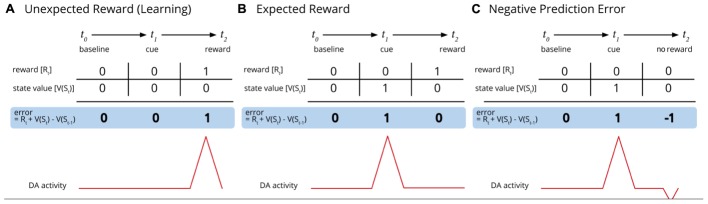
Cue-activated reward anticipation and prediction errors and subsequent dopaminergic activity (adapted from Keiflin and Janak, 2015). **(A)** Before the cue is conditioned, the unexpected reward results in phasic activation of dopamine neurons and a positive reward prediction error. **(B)** Once a reward is conditioned, the cue (and not the reward) results in a positive reward anticipation and increased dopamine activity. **(C)** When the cue occurs but is met without the expected award, the result is a negative prediction error and a reduction of dopamine activity below baseline.

**FIGURE 3:**
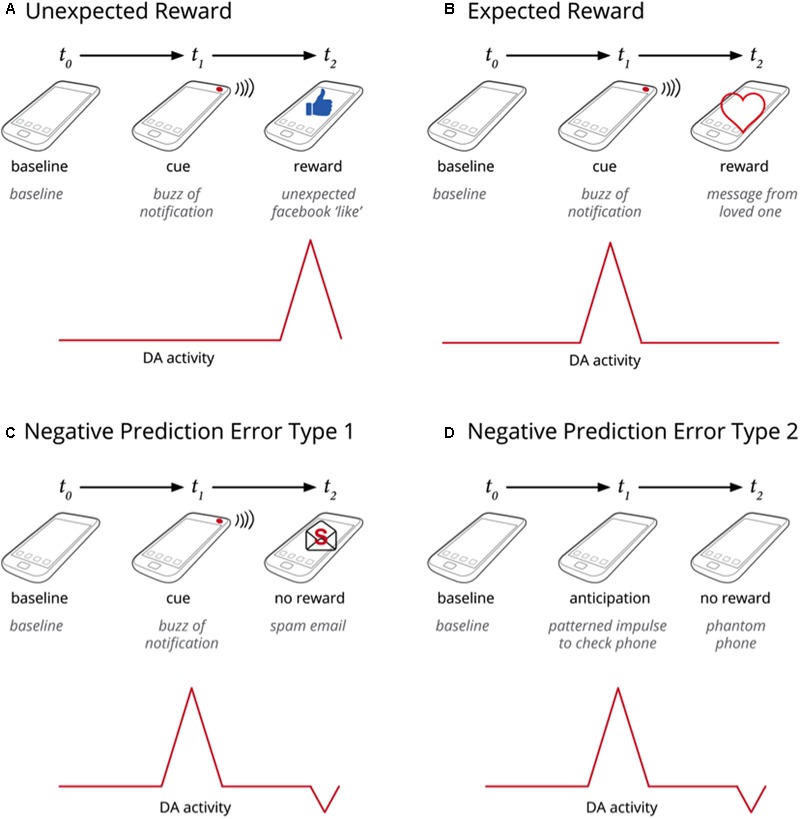
**(A–D)** Presents an extrapolation of the data presented in **Figure 2** to the present issue of smartphone addiction, whereby the dopamine activity increases at the anticipation of reward, and is reduced below baseline in cases where the expected reward is not met.

According to predictive-processing and free-energy theories of cognition, we do not immediately perceive the world as it is. Rather than directly respond to environmental stimulus, we first process information through our *expectations*. Immediate perception, in other words, first occurs through behavioral self-predictions modulated by prior experience (Friston and Kiebel, 2009; Ramstead et al., 2016). On this view, our brains generate statistical models of the world based on prior learning to provide us with predictions of what will arise in experience and how to act accordingly. In doing so our brains predict upcoming sensory states and compare them with actual sensory states, minimizing the differences between these distributions through constant updates of priors and actions (i.e., learning) (Ramstead et al., 2016, 2017). As our perceptual system constantly attempts to reduce uncertainty by computing abysmal amounts of disordered information to make it predictable, discrepancies between prediction and perception – *prediction errors* in the lingo – become commonplace. Cravings, on this view, could be conceptualized as prediction errors (Tobler et al., 2006) (**Figures 2, 3**).

As we mentioned above, associative learning and free-energy models can explain the pervasive expectation that the *anticipation* of smartphone notifications predicts an upcoming social reward. In turn, the intermittent schedule of smartphone notifications promotes stronger anticipations and more compulsive expectations, subsequently inducing prediction errors and affective disappointment.

Notifications are cues for checking behavior that eventually becomes habitual, even without the initial alert (Oulasvirta et al., 2012; Elhai et al., 2017). Recent studies reveal the magnitude of this habitual checking behavior, with the average individual spending over 3 h a day on their smartphone (Alter, 2017), tapping, typing, or swiping an average of 2617 times every day (dscout, 2016). The majority of users go on to experience prediction errors in the form of hallucinations that their phone is vibrating, a phenomenon entitled *phantom phone* (Sauer et al., 2015). These prediction errors reinforce habitual phone checking behaviors, which are a common gateway to smartphone addiction (Oulasvirta et al., 2012). Prediction errors can also occur in more subtle, but equally frequent and distressing way when precise patterned expectations are not met: a beep that we hope may be a message from a loved one or a Instagram ‘like’, for example, may turn out to be an incoming spam email or a message from one’s boss about an overdue task.

## The Dark Side of Social Monitoring?

Key models of ordinary cognition, like predictive processing, free-energy, associative learning, and social rehearsal, all offer clues to elucidate the newfangled phenomenon of smartphone addiction. We have seen that smartphone addiction harnesses basic human proclivities for social monitoring and associative learning. While we largely intend this paper to add a hopeful note about potentially healthy social causes of smartphone addiction amidst current panics, we cannot dismiss the growing consensus described above on such negative outcomes as depression, anxiety, and loneliness.

Smartphone use and depression are strongly correlated, and one causal theory suggests that smartphones, which are frequently used to access social networks, provide a platform for which to frequently (often negatively) compare oneself to others (Steers et al., 2014). We have argued, however, that social monitoring is a fundamentally normal – indeed necessary – part of ordinary human cognition. Classical evolutionary accounts of this propensity have emphasized the human fondness for gossip (Dunbar, 2004) and social comparison (Festinger, 1954) as conferring adaptive advantages to assess threats, track trends and shifts in others’ social status, and locate credible sources of cultural information and behavioral guides (Henrich, 2016). We add that comparing ourselves to others and against cultural norms also enables us to derive meaning, motivation, purpose, and a sense of identity. With socially connected smartphones, this evolutionary process simply runs on overdrive. We can now constantly and relentlessly engage in hyper-speed comparisons with social media content that is biased toward positivity. As media researchers have suggested, this continual stream of positive information about others allows users to repeatedly perform upward social comparisons and negative self-evaluations against a so-called “highlight reel” (Steers et al., 2014). Despite the obvious antigenic nature of cyber-mediated social comparisons, these accounts fail to acknowledge that the desire to socially connect is an even stronger motivator of smartphone use than the desire to do better than others.

To further address the non-benign concerns of smartphone overuse, the following section will once again employ theories of ordinary cognition to propose actions individuals can take to build happy, healthy relationships with mobile technology.

## Feeding Our Hungry Ghosts

If smartphone addiction rests on the fundamentally human proclivity toward prosociality, we can also learn to harness our social nature to pacify our cravings – or as Buddhic philosophies would put it, we can learn to sate our hungry ghosts.

In classical Buddhism, all creatures are said to undergo six life cycles, or go through six realms of existence (Levitt, 2003; Maté, 2008). They begin in Hell, where their life is described as constant torture, before moving on to the realm of Hungry Ghosts, where they are plagued by insatiable thirst, hunger, and cravings. Next comes the realm of Animals: a world of servitude and stupidity. This realm is followed by Asura, a world of anger, jealousy, and never-ending conflict. The Human realm comes next: a world of contradictions and indecisiveness; sweet and sour, hot and cold, happy and sad, good and evil. The human realm is a world of almost-thereness – wisdom and enlightenment are within reach, but never quite attained. Whether the next world of Deva-gati, or Heavenly Beings, offers final relief is open for debate (Levitt, 2003). It is world of intense pleasures, with intense miseries to match. Freedom from suffering, in the end, seems nowhere to be found. On a contemporary psychological reading, the Six Realms metaphor can also describe the quality and intentionality (aboutness) of the various states of consciousness and affect one will routinely encounter throughout the course of a day.

The Hungry Ghosts in this story can be understood as the state that regulates our cravings. This idea likely predates Buddhic philosophies, and is found in earlier Indian religions under the Sanskrit name *Preta* (Levitt, 2003). Pretas are supernatural creatures plagued by insatiable hunger and thirst. They have enormous stomachs, but very thin necks that can only support eating tiny things. In many Buddhist and Zen rituals, such as the *Oryoki* approach to eating and living, a single grain of rice is offered to Hungry Ghosts to acknowledge their existence and appease them a little (Levitt, 2003). The key here is to feed our Hungry Ghosts, and to find *just the right amount.* As we discuss further in our conclusion, this is consistent with harm-reduction approaches to addiction treatment that advocate responsible use over abstinence (Marlatt, 1996; Marlatt et al., 2011).

Recognizing smartphone cravings as Hungry Ghosts presents the opportunity to turn phone addiction into a intentional, just-enough ritual.

### Set Intentional Protocols

Many smartphone users feel trapped by their phones (Harmon and Mazmanian, 2013). The first step toward freedom from phone Hungry Ghosts, as we have seen, is to regain control of the pattern and make it predictable again. Switching off all sounds and notifications can help to ‘un-ring’ Pavlov’s proverbial bell and cull habitual checking behaviors. As we described above, smartphone addiction is mediated by the grasp of intermittent reinforcement schedules of social rewards. With this in mind, setting regular intervals to check one’s phone can reduce the strong cravings that arise from chaotic patterns of reward anticipation. When it comes to instant phone-mediated communication, we can also make our intentions and expectations transparent, and agree on protocols with others. Clear workplace communication policies, for example, those that prohibit evening and weekend emails, or setting clear expectation for time-windows in replying have been shown to be effective in reducing stress and increasing productivity (Mark et al., 2012). Similar ‘policies’ and clear expectations for when to text or not to text – what we call ‘intentional protocols’ – can be devised among friends, families and lovers.

## Conclusion

Like all natural proclivities, social monitoring and rehearsal can turn into Hungry Ghosts. The parallel with natural hunger and eating bear relevance to our argument about mobile technology. Blaming the rice, utensils, or kitchenware for one’s insatiable gluttony does not so much deflate the problem as miss the mark entirely. The root of addictions, as we have seen, is not in substances or rewards themselves, and much less in the technologies that deliver such rewards, but in the *anticipation* of rewards and in delivery schedules and rituals. The hard truth about cravings is that they are ultimately self-referential: cravings are about cravings first and foremost.

Smartphones and mobile technologies are not the root cause of modern distress. In post-industrial environments where foods are abundant and readily available, our cravings for fat and sugar sculpted by distant evolutionary pressures can easily go into insatiable overdrive and lead to obesity, diabetes, and rampant heart disease (Henrich, 2016; Harari, 2017). As we argued in this paper, the prosocial needs and rewards of a physically weak species that relied on collective parenting (Hrdy, 2009) and distributed knowledge (Tomasello, 2014; Henrich, 2016) to survive and carve a moral niche in a harsh world can similarly be hijacked to produce a manic theater of hyper-social monitoring. Smartphones may be equated to hyper-efficient kitchenware. Both technologies help optimize the processing and delivery of specific kinds of basic needs: food on the one hand, and social information on the other. The key to eating well and being good social beings lies in finding the quality and intensity of *consumption rituals*. As in the *oriyoki* ‘just the right amount’ hungry ghost feeding ritual, the recipe lies in setting appropriate intentions, quality of awareness, and pacing for the time, place, and amount of information, connection, and comparison one will consume. Turning off notifications, as we have seen, has been shown to help users regain control of when and why to check their devices intentionally (Alter, 2017). When used to judicious social ends, smartphone and social media use can yield many positive outcomes, from increased subjective well-being (Kim and Lee, 2011) to better romantic relationships (Steers et al., 2014).

To conclude, we recognize that there is a controversy in addiction research between abstinence-based and harm-reduction approaches (Marlatt, 1996; Marlatt et al., 2011). The latter approach, which we advocate in this article, supports safe and responsible use, and consideration of the complexities of the social context in which people are drawn to substance use. While recent studies have shown that temporarily giving up certain social media activities could increase subjective well-being (see Alter, 2017, for a review), the professional and social consequences of giving up smartphone use altogether are currently not known, and are likely to be costly in a age that requires instant connection in so many domains of social life.

Individuals, rather, can mobilize their intrinsic drive toward sociality to mitigate the negative and increase the positive effects of smartphone use. Pursuing healthy social connection is the antidote. Rather than use smartphones to compare our lives to the distorted slice of reality others present, we can use them as communication tools to foster genuine emotional relationships. When competitive comparison seems inevitable, we can subvert into a motivator or reminder of our own unique skills – or better yet, we can cultivate genuine joy for the achievements of others (Chandra, 2017).

## Author Contributions

SV provided the theoretical framework based on his previous work on cultural affordances and internet sociality. MS helped refine the theoretical framework and further ground it in neuroscience. SV and MS contributed equally to the writing.

## Conflict of Interest Statement

The authors declare that the research was conducted in the absence of any commercial or financial relationships that could be construed as a potential conflict of interest.

## Footnotes

1 “Helicopter parenting” is used as a derogatory term to describe obsessive parental supervision in most dimensions of children’s lives. Although the phrase first appeared in the l960′s (Ginott, 1965/2009), it is often said to characterize the post-1980s childrearing culture of “hovering around” one’s child. “Lawnmower parenting” (where one paves the way for children in all aspects of their lives), is sometimes used to describe more extreme forms of helicopter parenting. In November 2017, the Economist reported that parents in the United States and nine European countries (except for France), now spent 50% more time with their children than in 1965 (The Economist, 2017).
