# Corrigendum: Hypernatural Monitoring: A Social Rehearsal Account of Smartphone Addiction

**DOI:** 10.3389/fpsyg.2018.01118

**Published:** 2018-07-03

**Authors:** Samuel P. L. Veissière, Moriah Stendel

**Affiliations:** ^1^Department of Psychiatry, McGill University, Montreal, QC, Canada; ^2^Department of Anthropology, McGill University, Montreal, QC, Canada; ^3^Raz Lab in Cognitive Neuroscience, McGill University, Montreal, QC, Canada; ^4^Culture, Mind, and Brain Program, McGill University, Montreal, QC, Canada

**Keywords:** smartphone addiction, social neuroscience, evolutionary anthropology, predictive-processing, cultural affordances, social rehearsal, hungry ghosts

In the original article, there was a mistake in Figure 2 as published. The “reward” box in the upper right-hand corner of Panel C erroneously displayed a “1” instead of a “0”. The word “reward” below the “t_2_” frame in Panel C should have read “no reward.”

**Figure 2:**
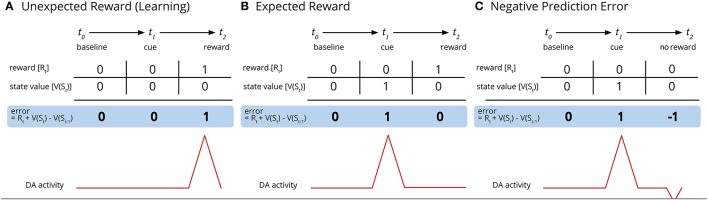
Cue-activated reward anticipation and prediction errors and subsequent dopaminergic activity (adapted from Keiflin and Janak, 2015). **(A)** Before the cue is conditioned, the unexpected reward results in phasic activation of dopamine neurons and a positive reward prediction error. **(B)** Once a reward is conditioned, the cue (and not the reward) results in a positive reward anticipation and increased dopamine activity. **(C)** When the cue occurs but is met without the expected award, the result is a negative prediction error and a reduction of dopamine activity below baseline.

The corrected Figure 2 appears below. The authors apologize for this error and state that this does not change the scientific conclusions of the article in any way.

The original article has been updated.

## Conflict of interest statement

The authors declare that the research was conducted in the absence of any commercial or financial relationships that could be construed as a potential conflict of interest.
